# Two new quill mite species of the genus *Psittaciphilus* Fain, Bochkov & Mironov, 2000 (Acariformes: Syringophilidae) associated with pigeons and doves (Columbiformes: Columbidae)

**DOI:** 10.1007/s11230-018-9822-0

**Published:** 2018-10-23

**Authors:** Katarzyna Kaszewska, Maciej Skoracki

**Affiliations:** 1 Department of Animal Morphology, Faculty of Biology, Adam Mickiewicz University in Poznań, Umultowska 89, 61-614 Poznan, Poland; 2 Laboratory and Museum of Evolutionary Ecology, Department of Ecology, Faculty of Humanities and Natural Sciences, University of Prešov, 17. novembra 1, 080 01 Prešov, Slovakia

## Abstract

Two new quill mite species of the genus *Psittaciphilus* Fain, Bochkov & Mironov, 2000 (Acariformes: Prostigmata: Syringophilidae) collected from columbiform birds (Columbiformes) are described: *Psittaciphilus montanus* n. sp. form the ruddy quail-dove *Geotrygon montana* Gosse from Trinidad and Tobago, Brazil and Panama, and *Psittaciphilus patagioenas* n. sp. from the band-tailed pigeon *Patagioenas fasciata* (Say) from Colombia and the scaled pigeon *Patagioenas speciosa* (Gmelin) from Surinam. A key to the species of the genus *Psittaciphilus* is provided. Our finding is the first record of the representatives of this genus on columbiform birds.

## Introduction

The quill mites of the family Syringophilidae Lavoipierre, 1953 (Acariformes: Prostigmata) are permanent and highly specialised ectoparasites, infesting quills of different types of feathers in the plumage of their avian hosts (Kethley, 1970). These mites show a high degree of host specificity, where most of species are mono- or stenoxenous parasites (Skoracki, 2011; Skoracki et al., 2016). Currently, the family includes 377 described species grouped in 62 genera and two subfamilies and recorded from about 500 host species belonging to 95 families and 24 orders (Zmudzinski & Skoracki, 2017).

The *Psittaciphilus* Fain, Bochkov & Mironov, 2000 is one of less known genera in the family Syringophilidae. Until now, only two species have been described, *P. amazonae* Fain, Bochkov & Mironov, 2000 and *P*. *fritschi* Fain, Bochkov & Mironov, 2000, both associated with parrots (Fain et al., 2000). Mites of this genus predominantly live inside the quills of the wing coverts, under-tail coverts and contour feathers (pers. obs.) and together with five other genera belong to the *Psittaciphilus*-generic-group established by Bochkov & Perez (2002).

In this paper we describe two new species of the genus *Psittaciphilus* associated with the South American pigeons and doves: *P. montanus* n. sp. from *Geotrygon montana* (Linnaeus) and *P. patagioenas* n. sp. from *Patagioenas fasciata* (Say) and *Patagioenas speciosa* (Gmelin).

## Materials and methods

The mite material used in the present study was collected from dry bird skins housed in the ornithological collection of the Bavarian State Collection of Zoology, Munich, Germany (ZSM). Feathers were examined using a dissecting microscope and opened with a fine scalpel. Before mounting, mites were softened and cleared in Nesbitt’s solution at room temperature for three days. Then, mites were mounted on slides in Hoyer’s medium. Taxonomic analysis of mite specimens was carried out with an Olympus BH-2 light microscope (Olympus Corp., Japan), equipped with DIC optics and a camera lucida. All measurements are given in micrometres. Measurements (ranges) of paratypes are given in parentheses following data for the holotype. In the descriptions below, the idiosomal chaetotaxy follows Grandjean (1939) as adapted for Prostigmata by Kethley (1990). The nomenclature of leg setae follows that proposed by Grandjean (1944). Morphological terminology follows Skoracki (2011). The scientific names of the birds follow Clements et al. (2017).

Specimen depositories are cited using the following abbreviations: AMU, A. Mickiewicz University, Department of Animal Morphology, Poznan, Poland; ZSM, Bavarian State Collection of Zoology, Munich, Germany.

**Family Syringophilidae Lavoipierre, 1953**

**Subfamily Syringophilinae Lavoipierre, 1953**

**Genus** ***Psittaciphilus*** **Fain, Bochkov & Mironov, 2000**

***Psittaciphilus montanus*** **n. sp.**

*Type-host*: *Geotrygon montana* (Linnaeus) (Columbiformes: Columbidae), ruddy quail-dove.

*Type-locality*: Trinidad Island, Aripo Berge, Trinidad and Tobago.

*Type-material*: Female holotype and 19 female paratypes from quills of under-tail coverts, 25.viii.1912, coll. Klages. The holotype and 17 paratypes were deposited in the AMU (Reg. no. AMU-SYR.571A); 2 female paratypes were deposited in the ZSM (Reg. no. ZSM20112080).

*Additional material examined*: Six females from quills of tail coverts of the same host species; Brazil, Para and Rio Negro, 1844, collector unknown; all mite specimens deposited in the AMU (Reg. no. AMU-SYR.571B). Eleven females from quills of tail coverts of the same host species; Panama, Chiriqui, 1895, coll. Dalmas; all mite specimens deposited in the AMU (Reg. no. AMU-SYR.571C), except 2 females in the ZSM (Reg. no. ZSM20112081).

*ZooBank registration*: To comply with the regulations set out in article 8.5 of the amended 2012 version of the *International Code of Zoological Nomenclature* (ICZN, 2012), details of the new species have been submitted to ZooBank. The Life Science Identifier (LSID) for *Psittaciphilus montanus* n. sp. is urn:lsid:zoobank.org:act:7E5C92C2-911D-474F-9BB0-B69DDCEBF06C.

*Etymology*: The name *montanus* is taken from the specific name of the host.

### Description (Fig. 1)

**Fig. 1:**
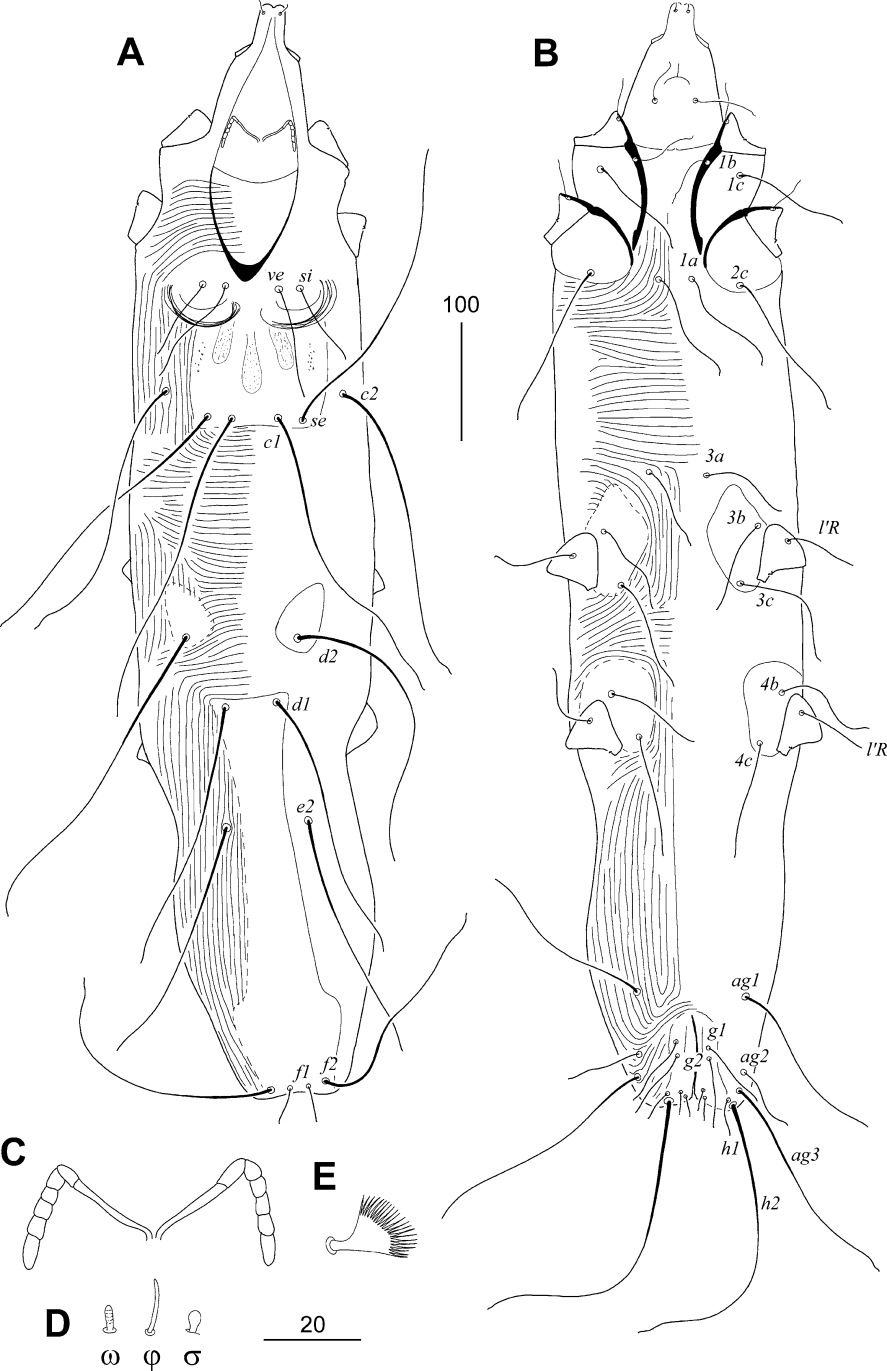
*Psittaciphilus montanus* n. sp., female. A, Dorsal view; B, Ventral view; peritremes; D, Solenidia of legs I; E, Fan-like seta *p′* of tarsi III

*Female*. Total body length of the holotype 600 (615–750 in 18 paratypes). *Gnathosoma*. Infracapitulum apunctate. Stylophore apunctate, 190 (160–190) long. Each medial branch of peritremes with 2 chambers, each lateral branch with 4 or 5 chambers (Fig. 1C). *Idiosoma*. Propodonotal shield sparsely punctate with 3 oval patches. Hysteronotal shield divided onto pair of oval sclerites surrounding bases of setae *d2*, and unpaired shield with bases of setae *d1* and fused to apunctate pygidial shield. Coxal fields I-IV apunctate. Setae *3a* situated out of coxal fields III. *Legs*. Solenidia of legs I as in Fig. 1D. Fan-like setae *p′* and *p″* of legs III and IV with 22–24 tines (Fig. 1E). Setae *tc″III*–*IV* 1.8–2.3 times longer than *tc′III*–*IV*. Setae *l′RIII* 1.2–1.3 times longer than *l′RIV*. *Lengths of setae*: *ve* 75 (60–85); *si* 60 (40–60); *se* 205 (210–245); *c1* 215 (220–230); *c2* 195 (180–205); *d1* 180 (170–190); *d2* 220 (230–255); *e2* 170 (170–195); *f1* 25 (30–35); *f2* 150 (150–180); *h1* 25 (25–30); *h2* 330 (310–315); *ag1* 125 (120–145); *ag2* 40 (35–55); *ag3* 165 (160–185); *g1* and *g2* 45 (30–45); *ps1* and *ps2* 10 (10); *tc′III*–*IV* 20 (15–30); *tc″III*–*IV* 40 (35–55); *l′RIII* 55 (55–60); *l′RIV* 45 (45); *3b* 60 (65–75); *3c* 75 (90–100). *Length ratios of setae*: *ve*:*si*:*se* 1.2–1.6:1:4–5.3; *d2*:*d1*:*e2* 1.3–1.4:1:1; *ag1*:*ag2*:*ag3* 2.4–3.1:1:3.2–4.1; *f1*:*f2* 1:5–6; *h1*:*f1* 1:1–1.2; *3b*:*3c* 1:1.2–1.3; *ps*:*g* 1:3–4.5.

Differential diagnosis

*Psittaciphilus montanus* n. sp. is morphologically similar to *P. fritschi*, collected from unidentified parrot from Zoo of Anverp (Belgium) (Fain et al., 2000). In females of both species, the stylophore, the infracapitulum and the pygidial shield are apunctate, and the medial branch of the peritremes has two chambers. This new species differs from *P. fritschi* by the following features: in females of *P. montanus* n. sp., each lateral branch of the peritremes has four or five chambers, the propodonotal shield is punctate and with three oval patches, and a pair of hysteronotal sclerites surrounding bases of setae *d2* is present. In females of *P*. *fritschi*, each lateral branch of the peritremes has six chambers, the propodonotal shield is apunctate and without oval shape patches, the hysteronotal shields surrounding bases of setae *d2* are absent.

***Psittaciphilus patagioenas*** **n. sp.**

*Type-host*: *Patagioenas fasciata* (Say) (Columbiformes: Columbidae), band-tailed pigeon.

*Type-locality*: Near San Juan River, Chocó Department Lama Hermosa, Colombia.

*Type-material*: Female holotype, 14 female paratypes from quill of under-tail coverts, 19.ix.1909, coll. Palmer. The holotype and 12 paratypes were deposited in the AMU (Reg. no. AMU-SYR.572); 2 female paratypes were deposited in the ZSM (Reg. no. ZSM20112082).

*Additional material examined*: Twenty-seven females from quills of wing coverts of the scaled pigeon *Patagioenas speciosa* (Gmelin) (Columbiformes: Columbidae); Surinam, Kraka, 23.ii.1963, coll. Haverschmidt; all mite specimens deposited in the AMU (Reg. no. AMU-SYR.573), except 2 females in the ZSM (Reg. no. ZSM20112083).

*ZooBank registration*: To comply with the regulations set out in article 8.5 of the amended 2012 version of the *International Code of Zoological Nomenclature* (ICZN, 2012), details of the new species have been submitted to ZooBank. The Life Science Identifier (LSID) for *Psittaciphilus patagioenas* n. sp. is urn:lsid:zoobank.org:act:4411F4FE-6BBF-45DE-8C62-5B82ED65A214.

*Etymology*: The name *patagioenas* is taken from the generic name of the host.

### Description (Fig. 2)

**Fig. 2:**
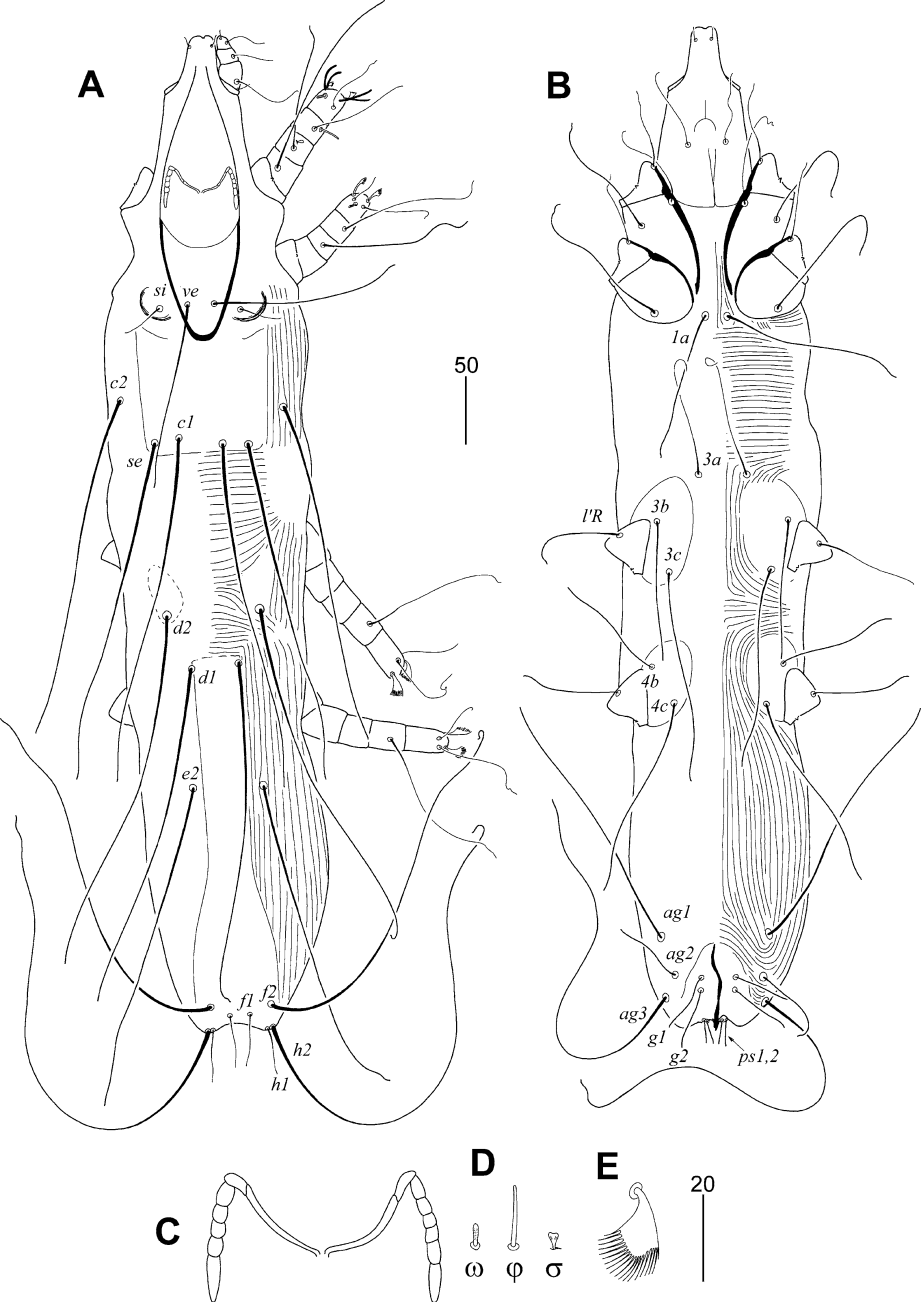
*Psittaciphilus patagioenas* n. sp., female. A, Dorsal view; B, Ventral view; peritremes; D, Solenidia of legs I; E, Fan-like seta *p′* of tarsi III

*Female*. Total body length of the holotype 735 (690–720 in 14 paratypes). *Gnathosoma*. Infracapitulum apunctate. Stylophore apunctate, 230 (220–230) long. Each medial branch of peritremes with 2 chambers, each lateral branch with 5 or 6 chambers (Fig. 2C). Propodonotal shield apunctate, without patches. Hysteronotal shield divided onto pair of oval sclerites surrounding bases of setae *d2*, and unpaired shield with bases of setae *d1* and fused to apunctate pygidial shield. Coxal fields weakly sclerotised and apunctate. Setae *3a* situated out of coxal fields III. *Legs*. Solenidia of legs I as in Fig. 2D. Fan-like setae *p′* and *p″* of legs III and IV with 19–20 tines (Fig. 2E). Setae *tc″III*–*IV* 2–2.3 times longer than *tc′III*–*IV*. Setae *l′RIII* and *l′RIV* subequal in length. *Lengths of setae*: *ve* 130 (120–125); *si* 40 (30–35); *se* 245 (245–260); *c1* 260 (260–285); *c2* 245 (235–250); *d1* 250 (240–250); *d2* 255 (260–280); *e2* (230–260); *f1* 45 (30–40); *f2* 280 (250–260); *h1* 30 (30–35); *h2* 360 (370–405); *ag1* 195 (190–210); *ag2* 70 (50–60); *ag3* 275 (280–295); *g1* and *g2* 45 (35–45); *ps1* and *ps2* 20 (10–15); *tc′III*–*IV* 35 (30–35); *tc″III*–*IV* 70 (70–80); *l′RIII* 55 (55–60); *l′RIV* 50 (50–70); *3b* 100 (70–95); *3c* 160 (130–145). *Length ratios of setae*: *ve*:*si*:*se* 3.4–4.3:1:6–8.5; *d2*:*d1*:*e2* 1:1:1; *ag1*:*ag2*:*ag3* 2.7–4.2:1:3.9–5.6; *f1*:*f2* 1:5.7–8.3; *h1*:*f1* 1:1; *3b*:*3c* 1:1.6–1.8; *ps*:*g* 1:2.2–3.5.

Differential diagnosis

*Psittaciphilus patagioenas* n. sp. is morphological similar to *P*. *amazonae*, collected from *Amazona amazonica* (Linnaeus) (Psittaciformes: Psittacidae) from Colombia (Fain et al., 2000). In females of both species, the stylophore and the infracapitulum are apunctate, the lengths of setae *ve* and *si* are 110–120 and 30–47 µm, respectively, and coxal fields I-IV are apunctate. This new species differs from *P. amazonae* by the following features: in females of *P. patagioenas* n. sp., the propodonotal and the pygidial shields are apunctate, the hysteronotal shields surrounding bases of setae *d2* are present, the lengths of hysteronotal setae *d1*, *d2*, and *e2* are 240–250, 255–280 and 230–260 µm, respectively. In females of *P*. *amazonae*, the propodonotal and the pygidial shields are punctate, the hysteronotal shields surrounding bases of setae *d2* are absent, and the lengths of hysteronotal setae *d1*, *d2*, and *e2* are 144–155, 179–192 and 179–184 µm, respectively.

**Key to the species of** ***Psittaciphilus***

1. Propodonotal shield punctate with three patches, setae *ve* 1.2–1.6 times longer than *si* ……………………………………………………………………………… *P*. *montanus* n. sp.
2. Propodonotal shield apunctate without patches, setae *ve* at least 2.4 times longer than *si* …………………………… 2
3. Lengths of setae *ve* and *si* 83–101 and 18–22 µm, respectively ………………………………………………….. *P*. *fritschi* Fain, Bochkov & Mironov, 2000
4. Lengths of setae *ve* and *si* 110–123 and 30–47 µm, respectively …………………………… 3
5. Propodonotal and pygidial shields punctate. Length of setae *d1*, *d2*, and *e2* 144–155, 179–192, and 179–184 µm, respectively. Hysteronotal shields surrounding bases of setae *d2* absent ………………………………………………… *P*. *amazonae* Fain, Bochkov & Mironov, 2000
6. Propodonotal and pygidial shields apunctate. Length of setae *d1*, *d2*, and *e2* 240–250, 260–280, and 230–260 µm, respectively. Hysteronotal shields surrounding bases of setae *d2* present …………………………………………………………………… *P*. *patagioenas* n. sp.
